# Anti-inflammation effects of the total saponin fraction from *Dioscorea nipponica* Makino on rats with gouty arthritis by influencing MAPK signalling pathway

**DOI:** 10.1186/s12906-020-03055-7

**Published:** 2020-08-25

**Authors:** Qi Zhou, Hui Juan Sun, Shu Min Liu, Xi Hong Jiang, Qiu Yue Wang, Shuang Zhang, Dong Hua Yu

**Affiliations:** 1 Research Institute of Chinese Medicine, Heilongjiang University of Chinese Medicine, Harbin, 150040 P.R. China; 2 Graduate School, Heilongjiang University of Chinese Medicine, Harbin, 150040 P.R. China; 3 Technological Innovation Team of Basic Theory Study Research of Institution of Higher Education in Heilongjiang Province, Heilongjiang University of Chinese Medicine, Harbin, 150040 P.R. China

**Keywords:** *Dioscorea nipponica* Makino, Gouty arthritis (GA), Mitogen-activated protein kinase (MAPK), Peroxisome proliferators-activated receptors (PPARs)

## Abstract

**Background:**

*Dioscorea nipponica* Makino is widely used in traditional Chinese medicine to treat gouty arthritis.

**Methods:**

Sixty male Wistar rats were divided into six groups: the normal group, model group, colchicine group (COL) and three total saponin groups (RDN) (high dose [160 mg/kg], middle dose [80 mg/kg] and low dose [40 mg/kg]). HE staining was used to detect the histopathologic changes of the synovial tissue of joint. Immunohistochemical method was used to detect the protein expressions of P-38, p-P38, JNK, p-JNK, ERK1/2, p-ERK1/2, MEK1/2, p-MEK1/2, MKK4, p-MKK4, ICAM1, VCAM1, and PPARγ in the synovial tissue of joint. Realtime PCR and WB methods were used to detect the mRNA and protein expressions of PPARγ and AdipoR2 in the synovial tissue of joint. The contents of CXCL1 and ADP in the blood serum were measured by Elisa method.

**Results:**

Our study showed that RDN could improve the situation of the synovial tissue, reduce the protein expressions of MKK4, p-MEK1/2, p-JNK, p-ERK1/2, ICAM1. They could also decrease the content of CXCL1 and increase the content of ADP in the blood serum.

**Conclusion:**

RDN has good effect of anti-inflammation. This is in part realized by influencing MAPK signalling pathway. It provides a new visual angle to reveal the mechanism of RDN to treat GA.

## Background

Gouty arthritis (GA) has recently been occurring more frequently in China and worldwide [1]. It is common in men compared to women [2]. This disorder results from high serum uric acid concentrations; monosodium urate (MSU) crystals are subsequently formed and deposited in both joints and tissues. Symptoms occur during acute episodes, and extreme pain is noted in this period [3]. Comorbidities are common in gout patients, such as arrhythmias, obesity, diabetes mellitus, and chronic kidney disease [4, 5]. Current drug used to treat GA may have some side effects which could be seen obviously from our previous study, such as stomach injury by indomethacin. It is urgent to identify new drugs to treat GA since it was detrimental to the health of the patients and especially in the condition of comorbidities [6]. Mitogen-activated protein kinases (MAPKs) play an important role during the pathogenesis of GA [7]. MAPKs are classified into main three types: extracellular signal-regulated kinase-1/2 (ERK1/2), c-Jun N-terminal kinases (JNKs) and p38 MAPKs. MAPKs activate the transcription factors NF-κB and AP-1 and regulate the expression of inflammatory factors, adhesion factors and chemokines. Peroxisome proliferator-activated receptors (PPARs) are useful in the treatment of a series of diseases such as type 2 diabetes mellitus, dyslipidaemia and cardiovascular diseases [8, 9]. Among them, PPARγ is especially relevant to GA. PPARγ competitively inhibits NF-κB and AP-1 activity. A PPARγ agonist has been effectively used to treat GA [10]. In addition, adiponectin (ADP) is a type of protein hormone that is secreted by fat cells. Its receptors are classified to three types, including AdipoR1, AdipoR2 and T-cadherin. ADP increases the sensitivity of insulin, exhibits anti-inflammatory effects, and protects the endothelium. PPARγ agonists promote increased expression of ADP and AdipoR2, which potentially serve as new targets to treat GA [11]. Drugs that are clinically used to treat GA exhibit various side effects. Gout drugs increase risk of the most common cancers, particularly in leukemia, non-Hodgkin’s, endometrial, breast and cervical cancer [12].. *Rhizoma Dioscorea Nipponicae* (RDN) is the rhizome of *Dioscorea nipponica* Makino that belongs to the Diocoreaceae family and is mainly distributed in the Northeast, North, West, and Qinghai regions of China. As an important traditional herb, this medicine has a long history of use in China [13]. This traditional medicine is utilized by a large number of individuals [14, 15]. Its major compound is steroidal saponin and RDN effectively reduces uric acid levels and exhibits anti-inflammatory effects. RDN is being explored as a new drug alone and in combination with other Western drugs in the treatment of GA [16, 17].

Our previous study showed that RDN could treat GA by interfering with the NALP3 inflammasome and the TLR2/4-IL-1R signal pathway, in this way, it influenced the expression and activities of inflammatory factors [18, 19]. Given that the MAPK-PPARγ is another branch down- stream signal pathway of TLR2/4-IL-1R that could influence the activities of transcriptional factors and thus regulates the expression of inflammatory factors, adhesion factors and chemokines, we assumed that it may also influence this signal pathway and had approved this by an in vitro study [20]. This study aims to further investigate the effect of RDN on interfering with the MAPK-PPARγ signalling pathway to treat GA by using animal models. In this study, we used MSU-induced Wistar rats as the GA models which was reported in a previously published paper [21]. HE staining was used to detect the histopathologic changes of the synovial tissue of joint in order to make sure the models were built successfully. The expression of the key protein factors involved in this signalling pathway by immunohistochemistry. PPARγ and Adipo2 mRNA and protein expression were also assessed by real-time PCR and WB methods. Serum chemokine (C-X-C motif) ligand protein CXCL1 and ADP levels were measured using the ELISA method. Our results showed that RDN reduces MKK4, p-MEK1/2, p-JNK, p-ERK1/2, and ICAM1 protein expression and increases PPARγ mRNA and protein expression. RDN also reduces serum CXCL1 levels and increases serum ADP levels. These results demonstrate that the MAPK-PPARγ signalling pathway is an effective target for the treatment of GA.

## Methods

### Chemicals and reagents

MSU was purchased from Sigma-Aldrich (St. Louis, USA), product No. was U 2875. ELISA test.

kits for CXCL1(DRE21465) and ADP (DRE 20701) were purchased from Tianjin Zombio Science and Technology Co., Ltd. (TianJin, China). Crude RDN crude drug extracts were purchased from the Harbin World Electuary Factory, product number was 090501. Colchicine (Purity rate > 99) was purchased from Xishuangbanna Pharmaceutical Co., Ltd. (Xishuangbanna, China, 151,121). RNA extraction (BK5106), reverse transcription (BK1401), and SYBR Green kits (BK1504) were purchased from Dalian Bao Biotechnology (Dalian, China). PMSF mother liquor (0754) and BSA (0332) were purchased fromAmresco (Washington, America). NC membrane was purchased from Millipore (Massachusetts, America).

### Plant material and extraction

RDN was purchased from the Heilongjiang Province Drug Company. The voucher specimens (hlj-201,508) of the herb were authenticated by Prof. Wang Zhen Yue, Hei long Jiang University of Chinese Medicine. One gram of crude drug was extracted with 6 mL 50% ethanol for 1.5 h for three times and purified by D-101 macroporous adsorption resin as described previously [18]. The extraction ratio was 4.97% (w/w).

### Determination of Rhizoma Dioscorea Nipponica content

The calculated content of RDN in the extract was approximately 55.9% [22].

### UPLC/MS analysis

UPLC/MS analysis was performed to chemically standardize the herbal extract.

Three major compounds in the RDN extract were identified: dioscin, protodioscin and pseudo protodioscin [22].

### Animals

We purchased sixty male Wistar rats (200 ± 20 g) from Shenyang Changsheng Biotechnology Co., Ltd. (China). They were raised in the animal house of the Institute of Chinese medicine, Heilongjiang University of Chinese Medicine and allowed 7 days to adapt to the environment before experiments.

Ten rats were housed per cage (600 × 450 × 280 mm) with the light on for 12 h and off for another 12 h each day, which was started since 07:00 a.m. They were housed at room temperature (23 ± 2 °C) with relative humidity (55 ± 5%) and given a standard chow and water ad libitum. To protect the animals, ethical approval for the experiments was obtained based on the Legislation on the Protection of Animals Used for Experiment Purposes (Directive 86/609/EEC). The Institutional Animal Care Committee approved the experiment (approval number was 20,190,921).

### Induction of gouty arthritis in rats using MSU

The method to induce rats of gouty arthritis were described in a previously published paper [18].

### Drug administration

Sixty male Wistar rats were divided into 6 groups randomly: The normal group, model group, groups treated with total saponin at a high dose (160 mg/kg), middle dose (80 mg/kg) and low dose (40 mg/kg) as well as colchicine group (COL) (0.3 mg/kg). The doses used was calculated according to clinical use [18]. All the drug groups were given every 24 h for 7 days. The normal group and the model group were administered normal saline as a control. MSU was used to induce the model of GA continuously for 5 days.

### Blood and synovial tissue sample collection

Whole blood and synovial samples were collected 1 hour after the model was induced on the seventh day as previously described. All efforts were made to minimize suffering. Before tissue collection, rats were deeply anesthetized by intraperitoneal injection of 3% pentobarbital sodium (45 mg/kg). At the end of the experiments, rats were sacrificed by cervical dislocation and synovial tissues were excised and kept in 4% paraformaldehyde and liquid nitrogen before tests [23].

### Histopathological assessment

Hematoxylin and eosin (HE) assessment to detect synovial tissue was described in a previously published paper [22].

### Immunohistochemical analysis of P-38, p-P38, JNK, p-JNK, ERK1/2, p-ERK1/2, MEK1/2, p-MEK1/2, MKK4, p-MKK4, ICAM1 and VCAM1

Rat synovial tissue was removed and post-fixed overnight in 4% paraformaldehyde. Then, tissues were embedded in paraffin. Serial sections (5 mm) were collected and immunohistochemically stained for P-38, p-P38, JNK, p-JNK, ERK1/2, p-ERK1/2, MEK1/2, p-MEK1/2, MKK4, p-MKK4, PPARγ, ICAM1 and VCAM1 using the high-pressure antigen repair method according the kit’s instructions. Primary antibodies included anti-rat P-38 antibody, anti-rat p-P38 antibody, anti-rat JNK antibody, anti-rat p-JNK antibody, anti-rat ERK1/2 antibody, anti-rat p-ERK1/2 antibody, anti-rat MEK1/2 antibody, anti-rat p-MEK1/2 antibody, anti-rat MKK4 antibody, anti-rat p-MKK4 antibody, anti-rat PPARγ antibody, anti-rat ICAM1 antibody and anti-rat VCAM1 antibody (all of these antibodies were raised in rabbit, 1:200). Each slide was examined at 200X magnification using an Olympus BX60 Microscope (Japan). All the antibodies used are described in Table 1 (Supplementary material).

**Table 1:** Summary of antibodies used in Immunohistochemical analysis and WB analysis

Company	Method	Description	Catalog number
Abcam	Immunohistochemical	P-38	ab27986
		p-P38	ab3838
		JNK	ab76125
		p-JNK	ab219584
		ERK1/2	ab17942
		p-ERK1/2	ab17942
		MEK1/2	ab131517
		p-MEK1/2	ab194754
		MKK-4	ab131494
		p-MKK-4	ab131494
		PPARγ	ab209350
		ICAM1	ab2213
		VCAM1	ab134047
Bioss	WB	PPARγ	BS-0530R
		AdipoR2	BS-0611R
Immunoway		GAPDH	YM3029

### Real-time PCR of PPARγ and AdipoR2

RNA was extracted using the classical TRIzol reagent method. The sequences of the gene-specific PCR primers and the lengths of the products are summarized in Table 2 (Supplementary material). Amplification was performed as previously described [18].

**Table 2:** Summary of Gene-Specific Real-Time PCR Primer Sequences

Description	Gene bank	Sense primer (5′-3′)	Anti-sense primer (3′-5′)
PPARγ	NM_013124	TAGGTGTGATCTTAACTGTCG	GCATGGTGTAGATGATCTCA
AdipoR2	NM_001037979	ATGTTTGCCACCCCTCAGTA	CAGATGTCACATTTGCCAGG
GAPDH	NM_008084	TGCACCACCAACTGCTTAC	GATGCAGGGATGATGTTC

### Western blotting of PPARγ and AdipoR2

Western blotting was performed as previously described [18]. The specific antibodies used included rPPARγ (1: 2000), rAdipoR2 (1:20000) and rGAPDH (1:2000) (Table 1).

### ELISA tests for CXCL1 and ADP

CXCL1 and ADP blood levels were assessed using kits.

### Statistical analysis

All data are expressed as the mean ± standard error of the mean (S.E.M.), and statistical analysis was performed using a one-way analysis of variance (ANOVA) followed by *Dunnett’s* t-test to determine the level of significance. A *P*-value < 0.05 was considered statistically significant.

## Results

### Histopathological analysis of rat synovial tissue

**Fig. 1:**
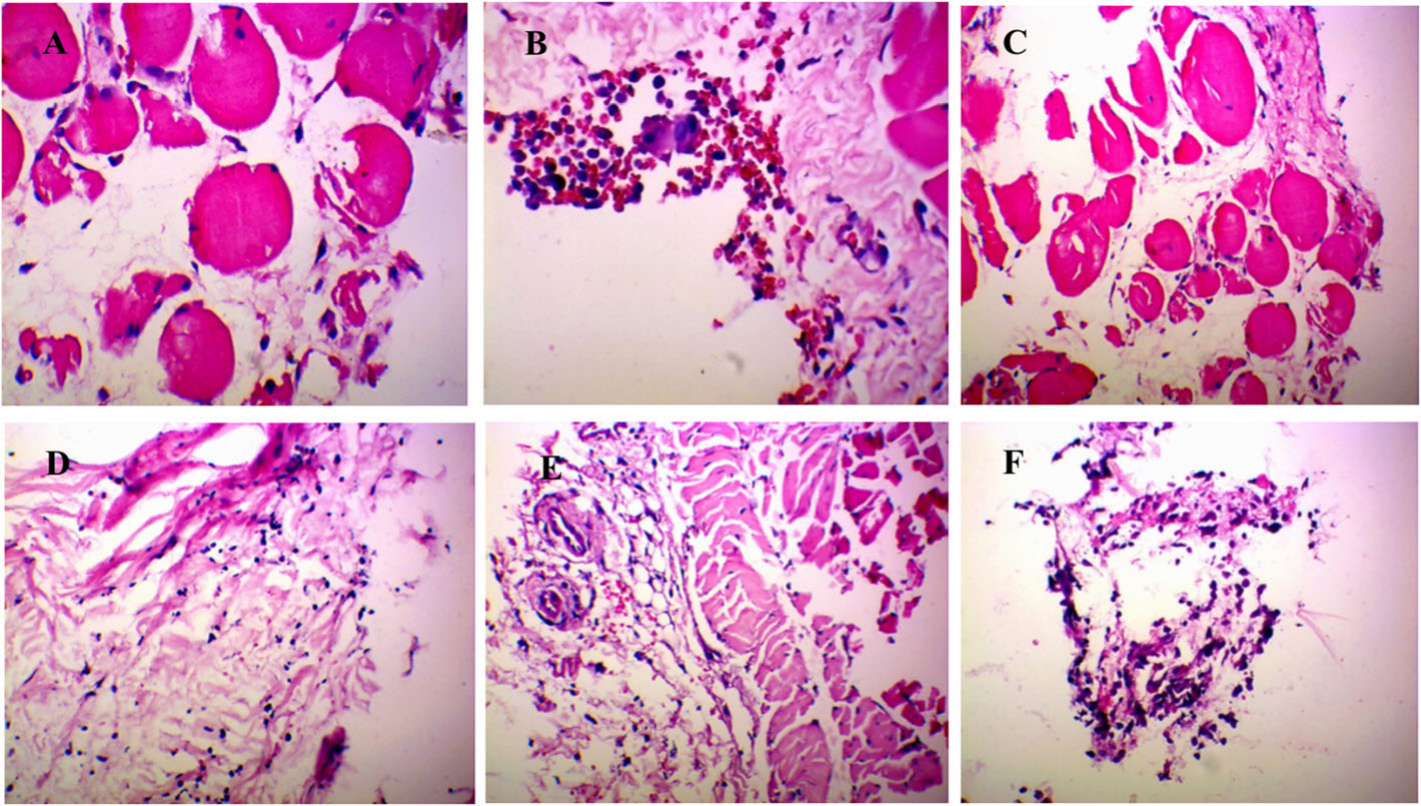
Histopathological analysis of rat synovium tissue. **a** Control group and **b** model group were treated with saline for 5 days. **c** High dose group **d** middle dose group and **e** low dose group were treated with extract of RDN for 5 days, dose amounted to 40 mg/kg, 80 mg/kg and 160 mg/kg daily, respectively. **f** COL group was treated with COL for 5 days. Each slide was examined at a magnification of 200 times. Histopathological changes of H&E-stained sections were used to assess the severity of GA, characterized by infiltration of inflammatory cells and synovium swelling and distention as well as vasocongestion and tissue necrosis. In contrast, healthy synovium showed almost no inflammatory cells

As shown in Fig. 1, synovial tissue was obtained from the knee joint 7 days after MSU injection. In the control group, synoviocytes formed a monolayer. No abnormal inflammatory cells were observed in synovial tissue. Light microscopic evaluation demonstrated a serious inflammatory reaction in GA rats. Specifically, synovial hyperplasia was obvious, and cells were irregularly arranged. In addition, inflammatory cell infiltration and pannus formation were observed. In contrast, synovial tissue damage in the RDN and COL groups was less severe. In addition, synovial hyperplasia was inhibited, and the inflammatory response was reversed.

### P38, p-P38, JNK, p-JNK, ERK1/2, p-ERK1/2, MEK1/2, p-MEK1/2, MKK4, p-MKK4, VCAM1 and ICAM1 protein expression as determined by Immunohistochemical analysis

**Fig. 2:**
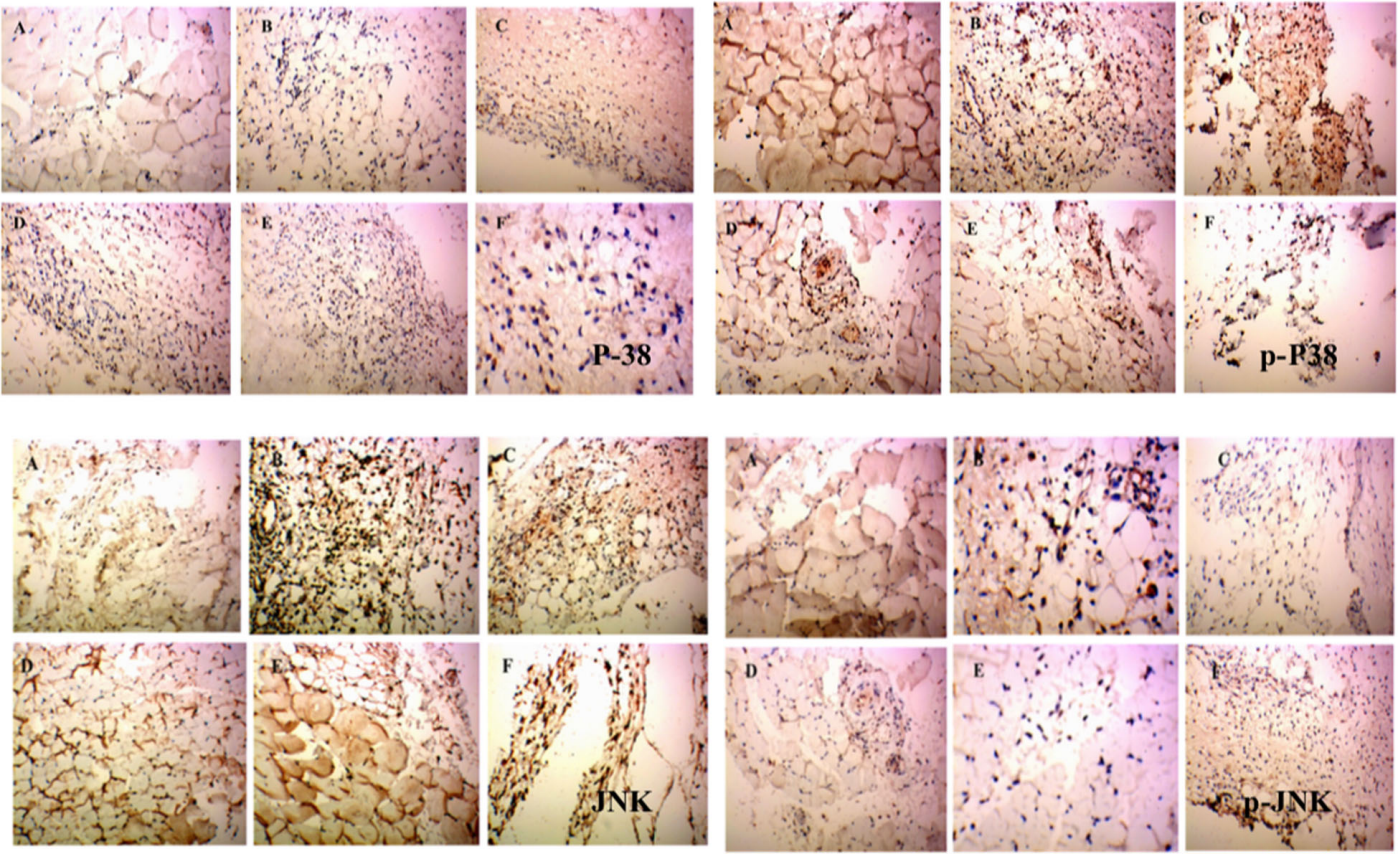
Immunohistochemical staining of P38, p-P38, JNK and p-JNK in rat synovium tissue. (**A**) Control group and (**B**) model group were treated with saline for 5 days. (**C**) High dose group (**D**) middle dose group and (**E**) Low group were treated with extract of RDN for 5 days, and dose amounted to 40 mg/kg, 80 mg/kg and 160 mg/kg daily, respectively. Each slide was examined at a magnification of 200 times and purple P38, p-P38, JNK and p-JNK positive cells can be found within rat synovium tissue. Images shown are a representative from three independent trials. ^###^*p* < 0.001 vs control group (independent samples *t*-test), ^***^p < 0.001 vs model group (one-way ANOVA followed by *Dunnett’s t*-test)

**Fig. 3:**
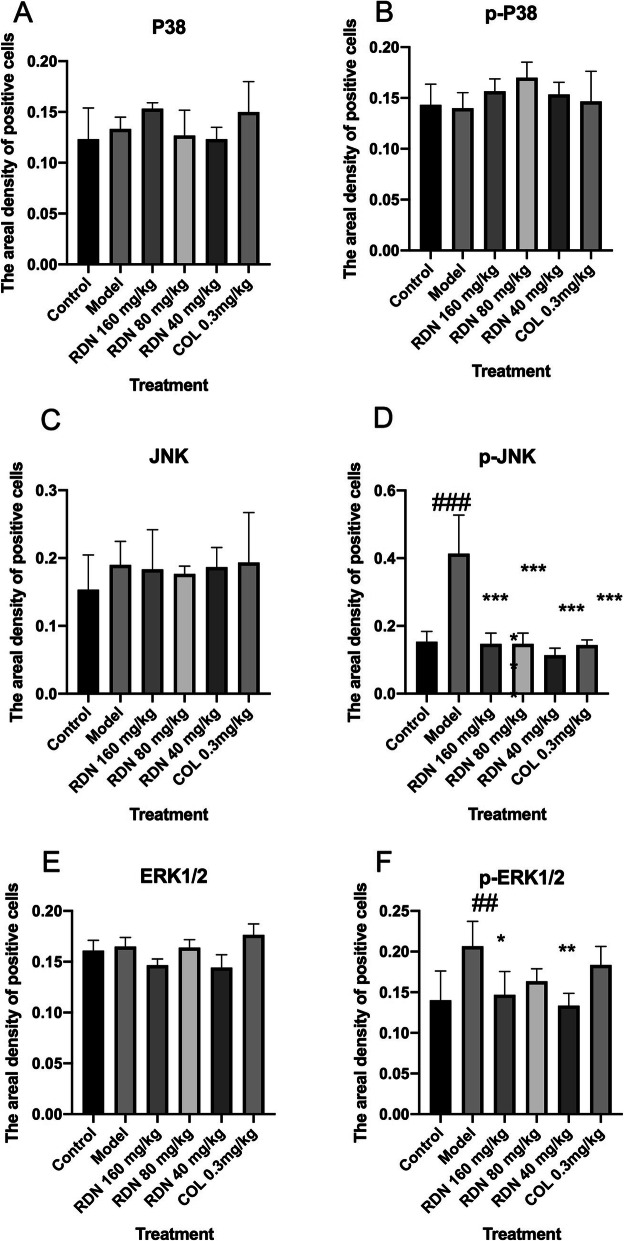
The levels of P38, p-P38, JNK, p-JNK, ERK1/2 and p-ERK1/2 in the rat synovium tissue. The areal densities of P38, p-P38, JNK, p-JNK, ERK1/2 and p-ERK1/2-positive cells in full image were calculated by Motic Med 6.0 Digital Medical Image Analysis System. Data represent the mean SE of each group (*n* = 10). ^##^*p* < 0.01 vs control group; ^###^*p* < 0.001 (independent samples *t*-test), ^*^p < 0.05 vs model group; ^**^p < 0.01 vs model group; ^***^p < 0.001 (one-way ANOVA followed by *Dunnett’s t*-test)

As shown in Figs. 2 and 3a, b and c, P38, p-P38 and JNK levels in the control group were 0.122 ± 0.017, 0.146 ± 0.021 and 0.153 ± 0.031, respectively. The respective levels in the model group were 0.134 ± 0.008, 0.141 ± 0.017 and 0.187 ± 0.017. No statistical differences were noted compared with control group. Compared with the model group, P38, p-P38 and JNK levels were 0.151 ± 0.004, 0.158 ± 0.013 and 0.185 ± 0.032 in the high-dose group; 0.123 ± 0.015, 0.170 ± 0.017, and 0.178 ± 0.007in the middle-dose group; 0.124 ± 0.006, 0.151 ± 0.012 and 0.188 ± 0.016 in the low-dose group; and 0.148 ± 0.017, 0.146 ± 0.029 and 0.193 ± 0.040 in the COL group. No statistically significant differences were noted compared with the model group.

As shown in Figs. 2 and 3d, p-JNK levels were 0.154 ± 0.015, 0.413 ± 0.064, 0.145 ± 0.019, 0.145 ± 0.019, 0.115 ± 0.010, and 0.143 ± 0.007 in the control, model, RDN high, RDN middle, RDN low and COL groups. Compared with control group, p-JNK protein levels were increased by 168.2% (*P* < 0.001) in the model group. Compared with model group, p-JNK protein levels were decreased by 64.9% (*P* < 0.001), 64.9% (*P* < 0.001), 72.2% (*P* < 0.001) and 65.4% (*P* < 0.001) in the RDN high, RDN middle, RDN low and COL groups.

**Fig. 4:**
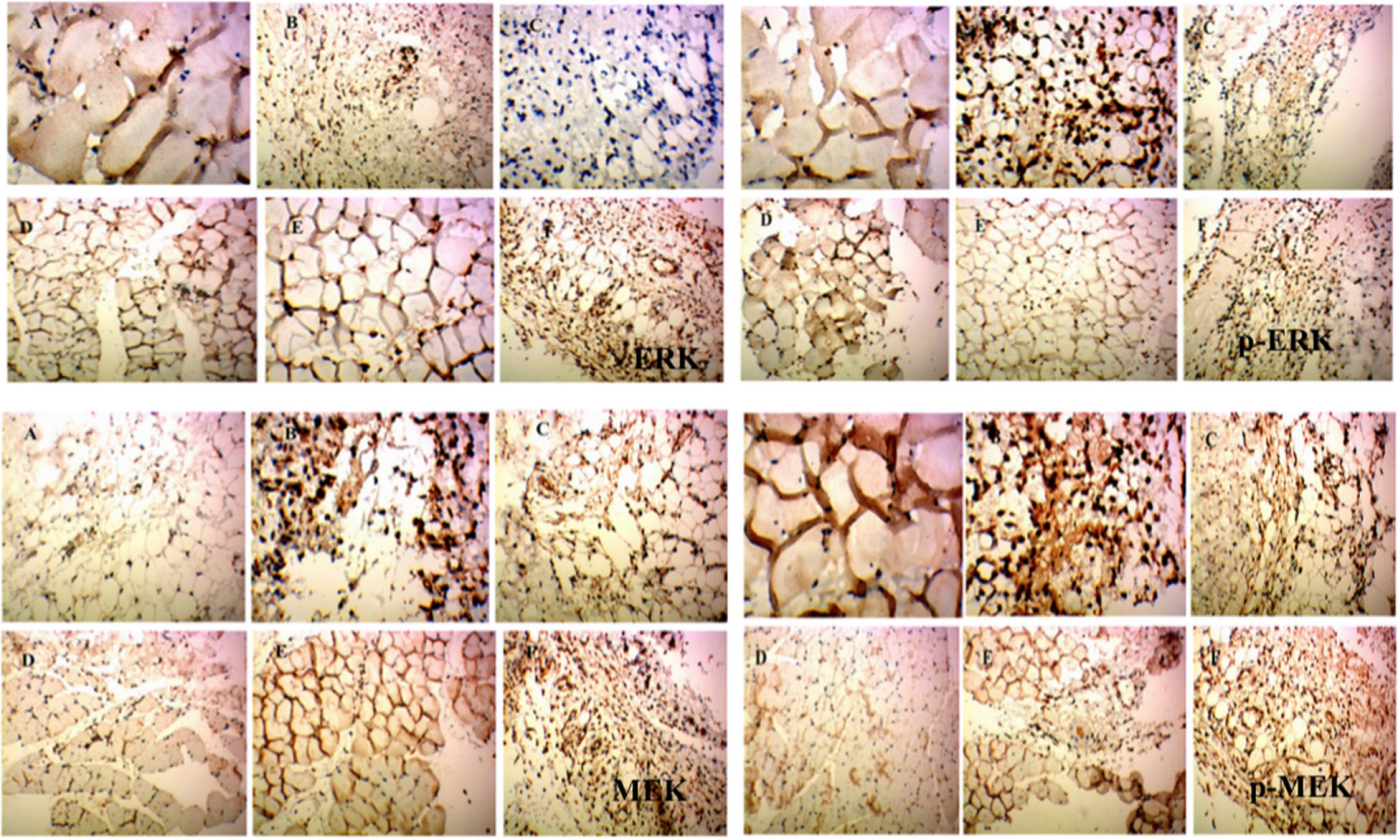
Immunohistochemical staining of ERK1/2, p-ERK1/2, MEK and p-MEK in rat synovium tissue. (**A**) Control group and (**B**) model group were treated with saline for 5 days. (**C**) High dose group (**D**) middle dose group and (**E**) Low group were treated with extract of RDN for 5 days, and dose amounted to 40 mg/kg, 80 mg/kg and 160 mg/kg daily, respectively. Each slide was examined at a magnification of 200 times and purple ERK1/2, p-ERK1/2, MEK and p-MEK positive cell can be found within rat synovium tissue. Images shown are a representative from three independent trials. ^##^*p* < 0.01 vs control group (independent samples t-test), *p < 0.05 vs model group; **p < 0.01 vs model group (one-way ANOVA followed by Dunnett’s t-test)

As shown in Figs. 3e and 4, ERK1/2 levels were 0.150 ± 0.22, 0.158 ± 0.012, 0.148 ± 0.017, 0.173 ± 0.028, 0.153 ± 0.011, and 0.189 ± 0.012 in the control group, model group, RDN high group, RDN middle group, RDN low group and COL group, respectively. No significant difference was noted among these groups.

As shown in Figs. 3f and 4, p-ERK1/2 levels were 0.141 ± 0.020, 0.207 ± 0.018, 0.143 ± 0.017, 0.164 ± 0.008, 0.131 ± 0.007, and 0.184 ± 0.012 in the control, model, RDN high, RDN middle, RDN low and COL groups, respectively. Compared with the control group, p-ERK1/2 protein levels were increased by 46.8% in the model group (*P* < 0.01). Compared with the model group, p-ERK1/2 protein levels were decreased by 30.9% (*P* < 0.05), 20.8, 36.7% (*P* < 0.01) and 11.1% in the RDN high, middle, low and COL groups.

**Fig. 5:**
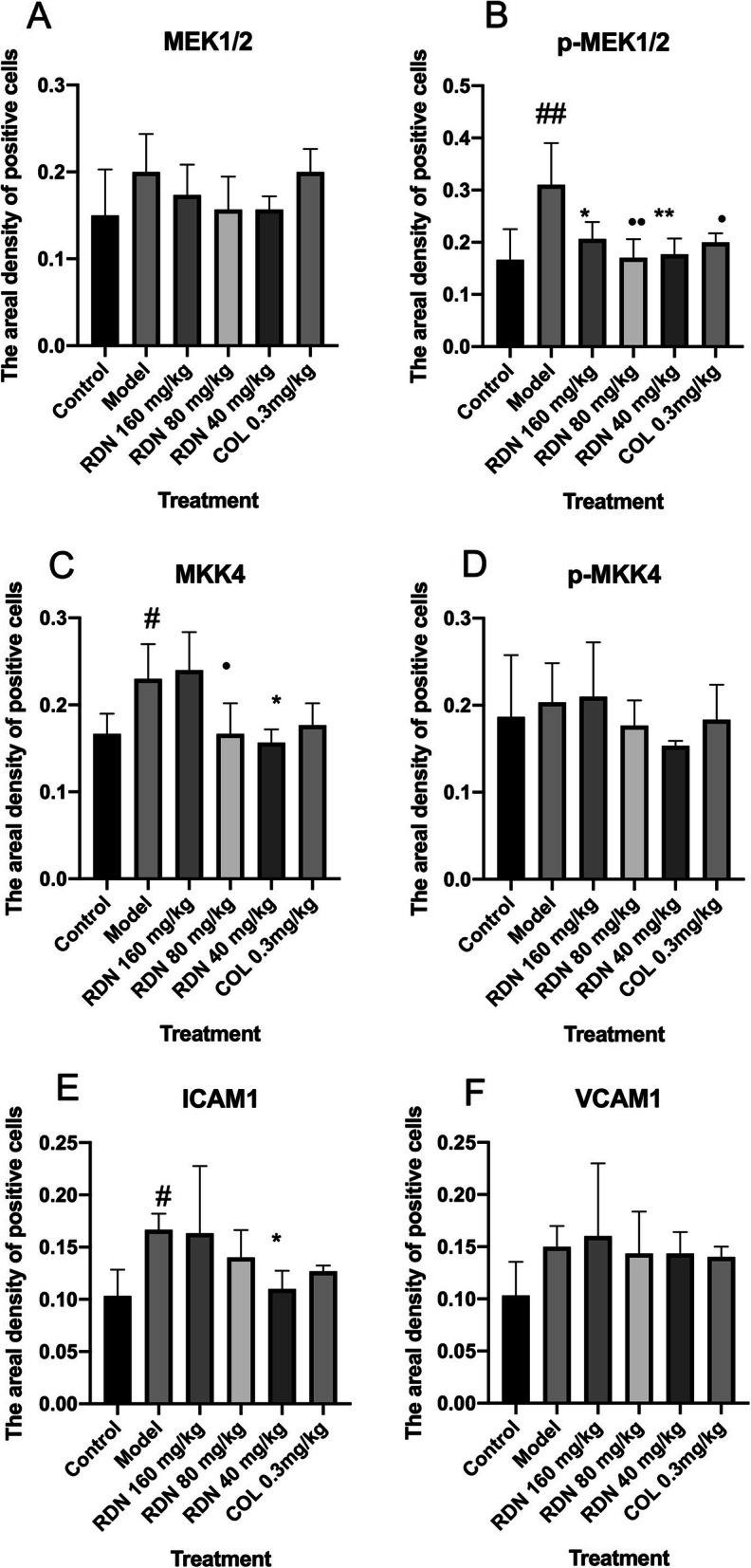
The levels of MEK1/2, p-MEK1/2, MKK4, p-MKK4, ICAM1 and VCAM1 in the rat synovium tissue. The areal densities of MEK1/2, p-MEK1/2, MKK4, p-MKK4, ICAM1 and VCAM1-positive cells in full image were calculated by Motic Med 6.0 Digital Medical Image Analysis System. Data represent the mean SE of each group (*n* = 10). ^#^*p* < 0.05 vs control group, ^##^*p* < 0.01 (independent samples *t*-test), ^*^p < 0.05 vs model group; ^**^p < 0.01 vs model group (one-way ANOVA followed by *Dunnett’s t*-test)

As shown in Figs. 4 and 5a, MEK1/2 levels were 0.149 ± 0.29, 0.201 ± 0.025, 0.174 ± 0.020, 0.154 ± 0.021, 0.159 ± 0.007, and 0.204 ± 0.014 in the control, model, RDN high, RDN middle, RDN low and COL groups, respectively. No significant differences were noted among all these groups.

As shown in Figs. 4 and 5b, p-MEK1/2 levels were 0.170 ± 0.033, 0.309 ± 0.046, 0.206 ± 0.019, 0.169 ± 0.022, 0.177 ± 0.016, and 0.198 ± 0.010 in the control, model, RDN high, RDN middle, RDN low and COL group. Compared with control group, p-MEK/2 protein levels were increased by 81.7% in the model group (*P* < 0.01). Compared with the model group, p-MEK1/2 protein levels were decreased by 33.3% (*P* < 0.05), 45.3% (*P* < 0.01), 42.7% (*P* < 0.01) and 35.9% (*P* < 0.05) in the RDN high, RDN middle, RDN low and COL groups.

**Fig. 6:**
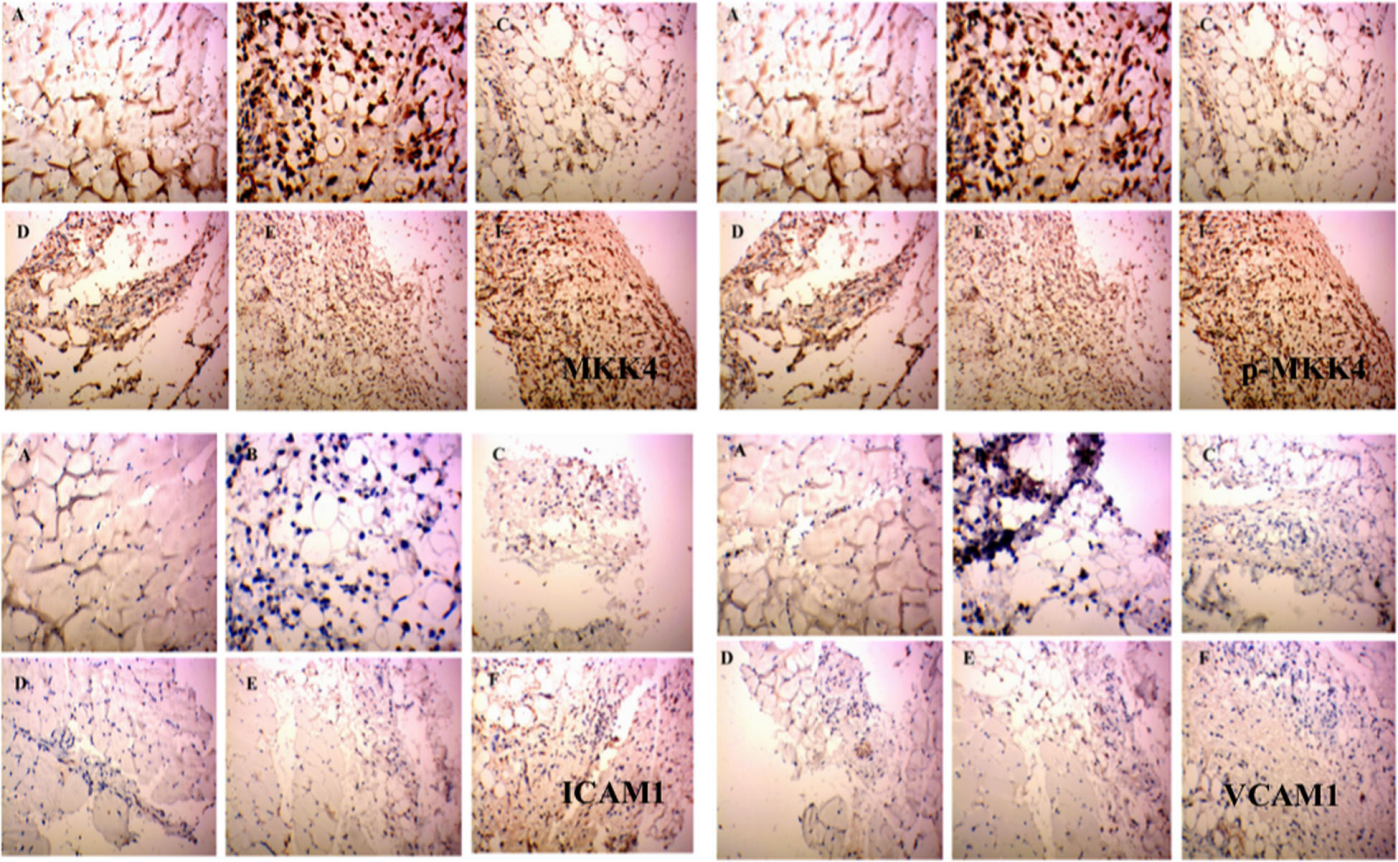
Immunohistochemical staining of MKK4, p-MKK, ICAM1 and VCAM1 in rat synovium tissue. (**A**) Control group and (**B**) model group were treated with saline for 5 days. (**C**) High dose group (**D**) middle dose group and (**E**) Low group were treated with extract of RDN for 5 days, and dose amounted to 40 mg/kg, 80 mg/kg and 160 mg/kg daily, respectively. Each slide was examined at a magnification of 200 times and purple MKK4, p-MKK, ICAM1 and VCAM1 positive cell can be found within rat synovium tissue. Images shown are a representative from three independent trials. ^#^*p* < 0.5 vs control group (independent samples t-test), *p < 0.05 vs model group (one-way ANOVA followed by *Dunnett’s t-test*)

As shown in Figs. 5c and 6, MKK4 levels were 0.165 ± 0.013, 0.229 ± 0.023, 0.236 ± 0.024, 0.157 ± 0.010, 0.179 ± 0.015, and 0.183 ± 0.009 in the control, model, RDN high, RDN middle, RDN low and COL groups. Compared with the control group, MKK4 protein levels were increased by 38.8% in the model group (*P* < 0.05). Compared with the model group, MKK4 protein levels were increased by 3.05% in the RDN high group. Compared with the model group, MKK4 protein levels were decreased by 31.4% (*P* < 0.05), 21.8% (*P* < 0.05) and 20.1% in the RDN middle, RDN low and COL groups.

As shown in Figs. 5d and 6, p-MKK4 levels were 0.185 ± 0.39, 0.201 ± 0.027, 0.207 ± 0.037, 0.175 ± 0.016, 0.153 ± 0.004, and 0.204 ± 0.014 in the control, model, RDN high, RDN middle, RDN low and COL groups. No significant differences were noted among all these groups.

As shown in Figs. 5e and 6, ICAM1 levels were 0.104 ± 0.014, 0.166 ± 0.006, 0.161 ± 0.038, 0.139 ± 0.013, 0.108 ± 0.006, and 0.128 ± 0.003 in the control, model, RDN high, RDN middle, RDN low and COL groups. Compared with the control group, ICAM1 protein levels wereincreased by 59.6% in the model group (*P* < 0.05). Compared with the model group, ICAM1 protein levels were decreased by 3.01, 16.3, 34.9% (*P* < 0.05), 35.9 and 20.1%in the RDN middle, RDN low and COL groups.

As shown in Figs. 5f and 6, VCAM1 levels were 0.102 ± 0.018, 0.149 ± 0.011, 0.159 ± 0.038, 0.144 ± 0.021, 0.143 ± 0.010, and 0.139 ± 0.006 in the control, model, RDN high, RDN middle, RND low and COL groups. No significance differences were noted among all these groups.

### Effects of RDN on PPARγ and AdipoR2 mRNA and protein expression

**Fig. 7:**
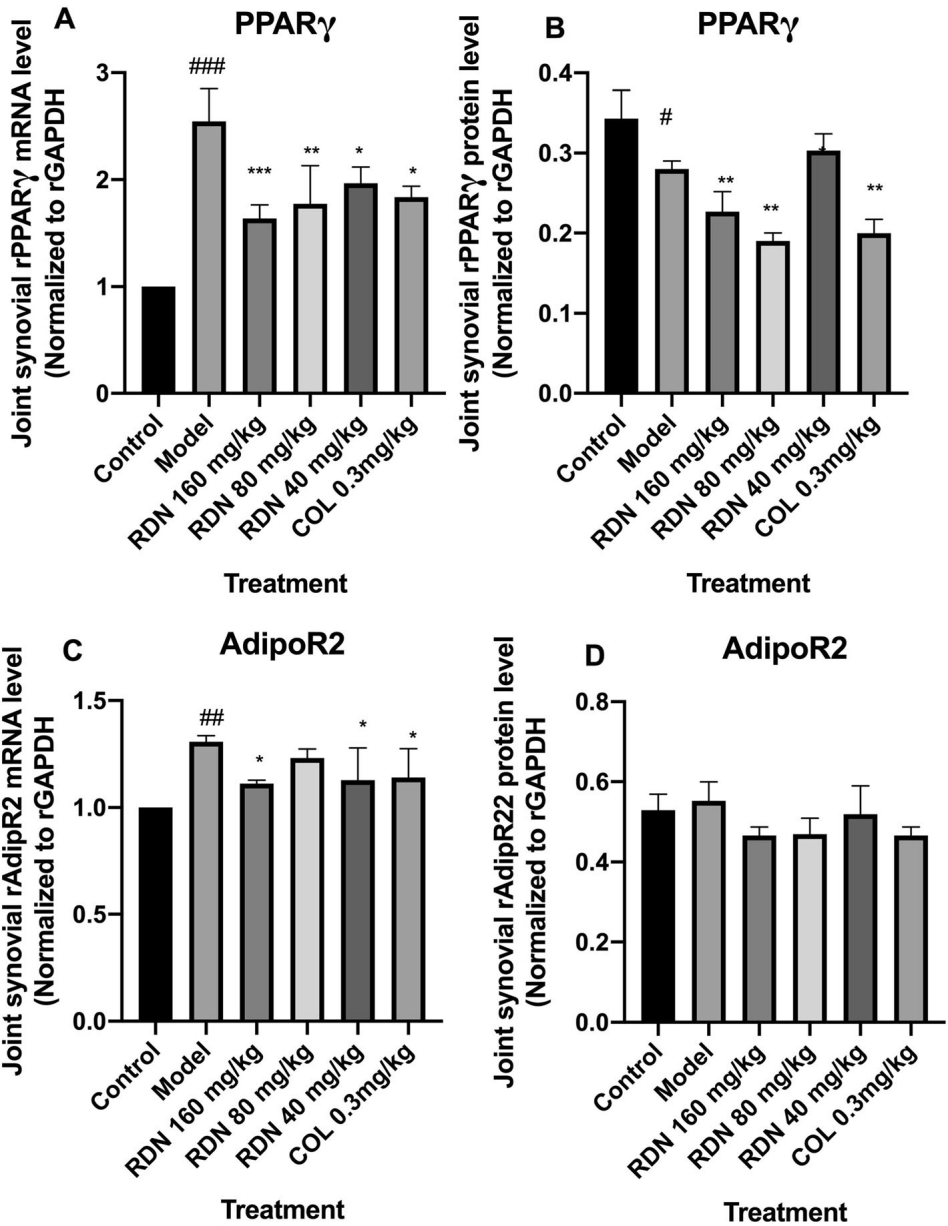
Effects of RDN and COL on mRNA and protein levels of PPARγ (**a** and **b**) and AdipoR2 (**c** and **d**) in the joint-synovial tissue in GA rats. The data represent the values of the mean ± S.E.M. for six mice. ^##^*P* < 0.01, ^###^*P* < 0.001 versus normal group (independent samples t-test). ^*^P < 0.05, ^**^P < 0.01, ^***^P < 0.001 versus model group (one-way ANOVA followed by *Dunnett’s t*-test)

**Fig. 8:**
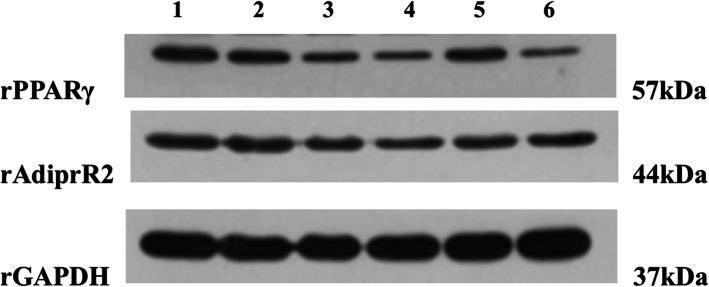
Effects of RDN and COL on the protein levels of rPPARγ and rAdipoR2 in the joint-synovial tissue of GA rats. RDN and COL were given 3 days before the models were induced every 24 h. The normal group and the model group were given normal saline at the same time. MSU solution was injected into both knees after intraperitoneal injection of 10% chloral hydrate to anaesthetize the rats an hour after the drugs were given since the third day. The drugs were given for 7 days continuously while the models were induced for 5 days altogether. 1.Normal group, 2. Model group,3. RDN high group, 4. RDN middle group, 5. RDN low group, 6. COL group

As shown in Table 3, Fig.7a, b, c, and d, and Fig. 8, Compared with the normal group, PPARγ mRNA expression was increased (*p* < 0.001), they were all decreased in the RDN high, middle, low dose groups and COL group (*p* < 0.001, *p* < 0.01, *p* < 0.05 and *p* < 0.05). Compared with the normal group, the protein expression in the model group was decreased significantly (*p* < 0.05). Compared with the model group, the expressions of PPARγ were decreased significantly in the high, middle and COL groups (*p* < 0.01, *p* < 0.01, and *p* < 0.01).

**Table 3:** Protein expression change of PPARγ and AdipoR2

Group	Dose (mg/kg)	PPARγ	AdipoR2
Normal	–	0.343 ± 0.020	0.528 ± 0.022
Model	–	0.560 ± 0.096^##^	0.553 ± 0.027
RDN	160	0.287 ± 0.008^**^	0.467 ± 0.011
RDN	80	0.213 ± 0.006^***^	0.469 ± 0.022
RDN	40	0.440 ±0.020	0.520 ± 0.040
COL	0.3	0.206 ± 0.008^***^	0.465 ± 0.012

Data represent mean ± SEM for10 rats, ^##^*p* < 0.01 compared with normal group (independent samples t-test); ^**^p < 0.01, ^***^p < 0.001 compared with model group (one-way ANOVA followed by Dunnett’s t -test)

Compared with the normal group, the mRNA expression of AdipoR2 in the model group was increased significantly (*p* < 0.01). Compared with the model group, the mRNA expression of AdipoR2 in the RDN high, low group and COL group were decreased significantly (*p* < 0.05, *p* < 0.05, and *p* < 0.05). However, there was no difference of AdipoR2 protein expression among these groups.

### Effects of RDN on CXCL1 and ADP levels in MSU crystal-induced rats

**Fig. 9:**
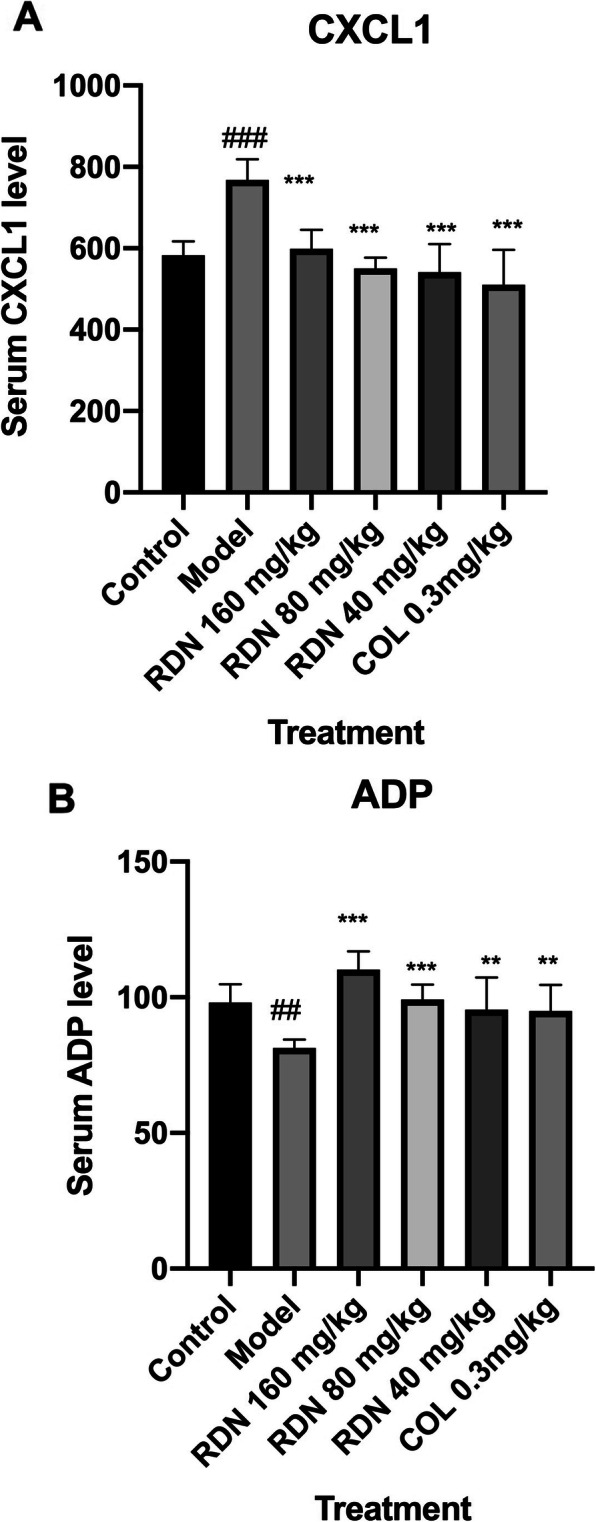
Effects of RDN and COL on the contents of CXCL1 and ADP in MSU crystal-induced rats. The data represent the values of the mean ± S.E.M. for ten rats. ^##^*P* < 0.01, ^###^*P* < 0.001 versus normal group (independent samples t-test). ^**^P < 0.01, ^***^P < 0.001 versus model group (one-way ANOVA followed by *Dunnett’s t*-test)

As shown in Fig. 9a and b, compared with the normal group, CXCL1 levels were increased significantly (*p* < 0.001) in the model group. The high (160 mg/kg) dose, middle (80 mg/kg), and low (40 mg/kg) RDN doses and COL dose significantly reduce CXCL1 levels (*p* < 0.001, *p* < 0.001, *p* < 0.001, and *p* < 0.001, respectively) compared with the model group. Compared with the normal group, ADP levels were decreased significantly (*p* < 0.01) in the model group. High (160 mg/kg), middle (80 mg/kg), and low (40 mg/kg) RDN doses and the COL dose significantly increase ADP levels (*p* < 0.001, *p* < 0.001, *p* < 0.01, and *p* < 0.01, respectively) compared with the model group.

## Discussion

GA is a disease that is associated with a difficult recovery, since its symptoms only manifest during the attack period and the chronic period when high concentrations of uric acid are present in the blood [5]. *Dioscorea nipponica* Makino is a traditional Chinese medicine that is clinically used to treat GA. However, the mechanism by which this drug acts against this disease is not completely understood. We have performed considerable research to reveal this mystery. Our previous study confirmed that the total saponins content of RDN was approximately 55%. Its main components, including dioscin, protodioscin, and pseudo protodioscin were identified by UPLC-MS [22]. In addition to its ability to reduce urate acid levels, it also has anti-inflammatory effects [18, 19, 22] Our newly published paper showed that RDN was effective in the treatment of GA by modulating the MAPK-PPARγ signaling pathway [24] However, it was an in vitro study that used fibroblast-like synoviocytes induced with IL-1β. The purpose of this study was to assess this mechanism using a GA rat model.

RDN regulates the abnormal expression of MKK4, p-MEK1/2, p-JNK, p-ERK1/2 and ICAM1 protein and PPARγ mRNA and protein in the synovial joint tissue. RDN reduced MKK4, p-MEK1/2, p-JNK, p-ERK1/2, and ICAM1 protein expression and decreased PPARγ mRNA and protein expression. RDN reduced serum CXCL1 levels and increases serum ADP levels showing that the MAPK signalling pathway is an effective target in GA. However, P38, p-P38, ERK1/2, JNK, p-MKK4 MEK1/2, VCAM1 and AdipoR2 protein expression levels did not obviously differ compared with the normal group.

MAPKs, including JNK, P38 and ERK1/2, are leading factors in GA attacks. Mitogen-activated protein kinase kinase4/7 (MKK4/7), mitogen-activated extracellular signal-regulated kinase1/2 (MEK1/2) and MKK3/6 are upstream kinases of JNK, ERK1/2 and P38, respectively [25–27]. In this study, RDN reduced the abnormal expression of MKK4, pMEK1/2, p-JNK and p-ERK1/2. This result is consistent with another study demonstrating that kinsenoside attenuates osteoarthritis by inhibiting the MAPK signalling molecules p-JNK, p-ERK and p-P38 [19].

A PPARγ agonist effectively inhibits the production of inflammatory factors in the treatment of GA rats [28]. A PPARγ agonist also promoted increased expressions of ADP and AdipoR2 [11]. In this study, PPARγ mRNA and protein expression is increased in the model group compared with the normal group. RDN decreased PPARγ mRNA and protein expression and increased serum ADP levels. RDN also decreas.ed. AdipoR2 mRNA levels; however, AdipoR2 protein levels did not differ compared with the control group. The result is quite consistent with our in vitro study [20]. This finding may be explained by the self-recovery features of GA. It showed that RRARγ is an RDN target that may have other mechanism that needs further investigation.

In GA attacks, various cell surface molecules are expressed. Among them, vascular cell adhesion molecule (VCAM)-1 and intercellular adhesion molecule (ICAM)-1 are critical [29]. When monosodium urate (MSU) induces monocyte and neutrophil granulocytes, VCAM1 activates these cells, and these activated cells enter tissues around the vasculature after adhering to vascular endothelial cells under the action of chemokines, such as C-X-C motif chemokine ligand (CXCL)1. In this study, RDN reduced expression of both ICAM1 and CXCL1; however, similar VCAM1 protein levels were noted in the model and control groups. It was also very interesting that the low dose of RDN showed better effect compared with the high dose group. As there were multi-components and multi-targets of RDN to act in this process, when one target was achieved by supersaturation, the mechanism may be changed in some aspect.

Our results showed that RDN exhibits anti-inflammatory effects by modulating the MAPK/PPARγ signalling pathway further confirming our previous study. Since GA is a disease caused by high serum uric acid concentrations, hypertension, hyperlipidaemia and other diseases that are caused by the same mechanisms often occur as complications [30]. Chinese medicine often targets multiple targets since it has multiple components. This study showed that the MAPK/PPARγ signalling pathway is a useful target in the treatment of GA. Thus, RDN should be explored as a new drug to treat GA and, especially, its complications. However, further studies to knock down or over express the key genes of this signalling pathway should be done to verify the above results.

## Conclusion

RDN is potentially useful in the treatment of GA by modulating the MAPK/PPARγ signalling pathway. This study provides a pharmacological foundation to explore RDN as a new medicine to treat GA.

## Supplementary information

**Additional file 1.**

## Data Availability

The datasets used and analysed during the current study are available from the corresponding author upon reasonable request.

## Abbreviations

GA: Gouty arthritis
MAPK: Mitogen-activated protein kinase
PPARs: Peroxisome proliferators-activated receptors
RK1/2: Extracellular signal regulated kinase 1/2
JNK: c-Jun N-terminal kinase
NF-κB: Nuclear factor kappa B
MKP: MAPK phosphatase
CXCL1: C-X-C motif chemokine1
VCAM-1: Vascular cell adhesion molecule1
ICAM1: Intercellular adhesion molecular1
