# Supplementary material for: Anti-inflammation effects of the total saponin fraction from Dioscorea nipponica Makino on rats with gouty arthritis by influencing MAPK signalling pathway
Source: BMC Complement Med Ther. 2020 Aug 25;20:261. doi: 10.1186/s12906-020-03055-7 (PMC7446153; doi:10.1186/s12906-020-03055-7)
Supplement: Supplementary file 1 — Additional file 1. [file 12906_2020_3055_MOESM1_ESM.docx]

**1 2 3 4 5 6**

**rPPARγ
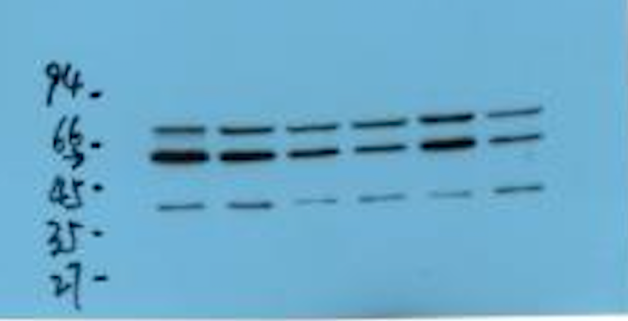
 57kDa**

**rGAPDH**
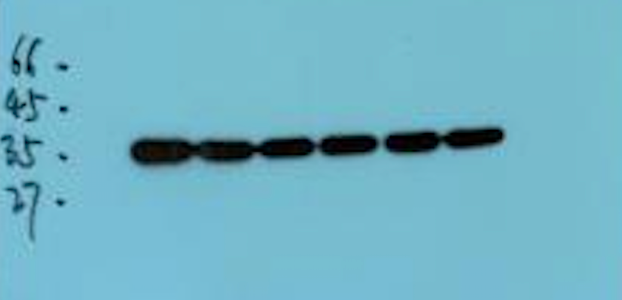
 **37kDa**

**rAdiprR2
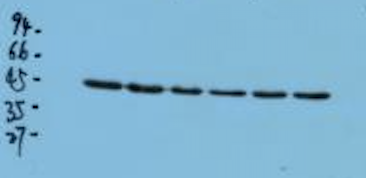
 44kDa**

**rGAPDH**
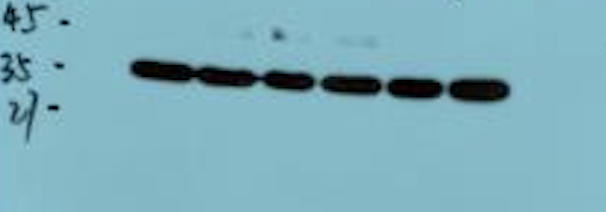
 **37kDa**

Fig.11. Effects of RDN and COL on the protein levels of rPPARγ and rAdipoR2 in the joint-synovial tissue of GA rats. RDN and COL were given three days before the models were induced every 24 h. The normal group and the model group were given normal saline at the same time. MSU solution was injected into both knees after intraperitoneal injection of 10% chloral hydrate to anaesthetize the rats an hour after the drugs were given since the third day. The drugs were given for seven days continuously while the models were induced for five days altogether. 1.Normal group, 2. Model group,

3. RDN high group, 4. RDN middle group, 5. RDN low group, 6. COL group.
